# Examining virtual driving test performance and its relationship to individuals with HIV-associated neurocognitive disorders

**DOI:** 10.3389/fnins.2022.912766

**Published:** 2022-08-24

**Authors:** David Grethlein, Vanessa Pirrone, Kathryn N. Devlin, Will Dampier, Zsofia Szep, Flaura K. Winston, Santiago Ontañón, Elizabeth A. Walshe, Kim Malone, Shinika Tillman, Beau M. Ances, Venk Kandadai, Dennis L. Kolson, Brian Wigdahl

**Affiliations:** ^1^Diagnostic Driving, Inc., Philadelphia, PA, United States; ^2^Department of Computer Science, The Games Artificial Intelligence and Media Systems (GAIMS) Center, College of Computing and Informatics, Drexel University, Philadelphia, PA, United States; ^3^Department of Microbiology and Immunology, College of Medicine, Institute for Molecular Medicine and Infectious Disease, Drexel University, Philadelphia, PA, United States; ^4^Applied Neuro-Technologies Lab, Department of Psychological and Brain Sciences, College of Arts and Sciences, Drexel University, Philadelphia, PA, United States; ^5^Division of Infectious Diseases and HIV Medicine, Department Medicine, Partnership Comprehensive Care Practice, College of Medicine, Drexel University, Philadelphia, PA, United States; ^6^Center for Injury Research and Prevention, Children’s Hospital of Philadelphia, Philadelphia, PA, United States; ^7^Department of Pediatrics, Perelman School of Medicine, University of Pennsylvania, Philadelphia, PA, United States; ^8^College of Medicine, Drexel University, Philadelphia, PA, United States; ^9^Department of Neurology, Hope Center for Neurological Disorders, School of Medicine, Washington University, St. Louis, MO, United States; ^10^Department of Neurology, Perelman School of Medicine, University of Pennsylvania, Philadelphia, PA, United States

**Keywords:** HIV-associated neurocognitive disorders, driving simulator, screening tool, variable selection, impairment detection

## Abstract

**Significance:**

Existing screening tools for HIV-associated neurocognitive disorders (HAND) are often clinically impractical for detecting milder forms of impairment. The formal diagnosis of HAND requires an assessment of both cognition and impairment in activities of daily living (ADL). To address the critical need for identifying patients who may have disability associated with HAND, we implemented a low-cost screening tool, the Virtual Driving Test (VDT) platform, in a vulnerable cohort of people with HIV (PWH). The VDT presents an opportunity to cost-effectively screen for milder forms of impairment while providing practical guidance for a cognitively demanding ADL.

**Objectives:**

We aimed to: (1) evaluate whether VDT performance variables were associated with a HAND diagnosis and if so; (2) systematically identify a manageable subset of variables for use in a future screening model for HAND. As a secondary objective, we examined the relative associations of identified variables with impairment within the individual domains used to diagnose HAND.

**Methods:**

In a cross-sectional design, 62 PWH were recruited from an established HIV cohort and completed a comprehensive neuropsychological assessment (CNPA), followed by a self-directed VDT. Dichotomized diagnoses of HAND-specific impairment and impairment within each of the seven CNPA domains were ascertained. A systematic variable selection process was used to reduce the large amount of VDT data generated, to a smaller subset of VDT variables, estimated to be associated with HAND. In addition, we examined associations between the identified variables and impairment within each of the CNPA domains.

**Results:**

More than half of the participants (*N* = 35) had a confirmed presence of HAND. A subset of twenty VDT performance variables was isolated and then ranked by the strength of its estimated associations with HAND. In addition, several variables within the final subset had statistically significant associations with impairment in motor function, executive function, and attention and working memory, consistent with previous research.

**Conclusion:**

We identified a subset of VDT performance variables that are associated with HAND and assess relevant functional abilities among individuals with HAND. Additional research is required to develop and validate a predictive HAND screening model incorporating this subset.

## Introduction

In the era of antiretroviral therapy, the most severe form of HAND (HIV-associated dementia) is rare (∼2% prevalence). Although less severe, functional impairment associated with milder forms of HAND (∼15–60% prevalence in PWH) is more common (Saylor et al., 2016; World Health Organization, 2017; Yusuf et al., 2017). Failure to detect HAND early is associated with a lower quality of life and decreased survival (Heaton et al., 2004; Antinori et al., 2013; Vance et al., 2014; Kronemer et al., 2017). Earlier detection of HAND through implementation of screening tools in the outpatient clinic is a diagnostic goal worldwide; however, previously tested screening tools are limited in their sensitivity and specificity to detect these milder forms of HAND (Roebuck-Spencer et al., 2017).

In part, due to new reimbursable billing codes, administration of cognitive screening tests have emerged (Abraham, 2013; Federal Register., 2015; Roebuck-Spencer et al., 2017); however, significant limitations in these tools for HAND have been identified (Roebuck-Spencer et al., 2017). The existing tools (1) are not self-directed and require skilled staff for administration and interpretation (commonly not found outside of research protocols) (Antinori et al., 2013); (2) lack face validity to assess common functional activities of daily living (ADL) (Antinori et al., 2013); and (3) have failed to meet acceptability for sensitivity and specificity and have limited value for detecting milder forms of HAND [e.g., IHDS (Sacktor et al., 2005; Chan et al., 2019), MoCA (Rosca et al., 2019), MMSE (de Souza et al., 2016), SSQ (de Souza et al., 2016), and CAT-Rapid (Antinori et al., 2007, 2013)]. Additionally, the lack of ecological validity of current screening tools limits their value beyond screening: these tools provide limited insight on ADLs and therefore, cannot provide practical guidance on addressing potential impairment (Antinori et al., 2013). Thus, there is a need for screening tools that are rapid, cost-effective, and reliable for detecting milder forms of HAND, while providing practical guidance on ADL.

Driving is cognitively demanding (Karthaus and Falkenstein, 2016) and an instrumental ADL (Green Car Reports, 2014; Brenan, 2018), which we hypothesize, can serve as a platform for the earlier detection of milder forms of HAND. Safe driving requires efficient multisensory processing and integration, motor control of the vehicle, sustained attention, and related cognitive abilities to manage distractions, maintain good situational awareness, and to make quick decisions and rapid responses in critical moments to avoid collisions (Wadley et al., 2009; Walshe et al., 2017). Thus, driving requires many of the cognitive abilities typically affected by HAND, such as complex attention, executive function, and fine motor speed and dexterity (Roenker et al., 2003; Wadley et al., 2009; Karthaus and Falkenstein, 2016; Walshe et al., 2017). Previous studies have demonstrated impaired driving performance among those with HAND: PWH with cognitive impairments had slower reaction times, as compared to normative controls (Vance et al., 2014), increased time requirements for completion of the driving simulation task (Foley et al., 2013), impaired route planning (Foley et al., 2013), and an increased risk for traffic collisions (Marcotte et al., 1999, 2004; Gouse et al., 2021). However, previous applications of driving assessments in PWH were not scalable for routine clinical settings, as they utilized on-road evaluations or laboratory-grade equipment.

In a novel application, this research program aims to utilize a well-established virtual driving test (VDT) platform (Walshe et al., 2020) that has been validated to predict on-road performance (Winston et al., 2019; Grethlein et al., 2020) as a probe to screen for neurocognitive impairment among PWH. The currently used HAND screening tools (e.g., IHDS, MoCA, MMSE, SSQ, CAT-Rapid) may have minimal direct costs associated with them (e.g., cost to purchase license for use, cost to purchase equipment, if any); however, there is an indirect cost of administering these tests in routine clinical practice (e.g., trained staff labor). We do not argue that the VDT would be any less expensive than existing tools; however, our experience of using the VDT as a platform to assess safe driving skills in the commercial market demonstrates that our delivery costs for the VDT is less than $2.00/test (inclusive of hardware, administration, and software license) when used at scale.

Given that the VDT platform is commercially used as a rapid and low-cost assessment to test complicated tasks associated with safe driving, it has the potential to also be used to screen for HAND (using driving as a probe). As a first step in evaluating the potential for the VDT to be used as a HAND screening tool, this preliminary study aimed to (1) evaluate whether VDT performance variables were associated with a HAND diagnosis and if so; (2) systematically identify a manageable subset of variables for use in a future screening model for HAND. As a secondary objective, we examined the relative associations of identified variables with impairment within seven individual domains used to diagnose HAND, to better understand the impact of impairment on a highly common ADL.

## Materials and methods

### Study participants and eligibility criteria

In a cross-sectional design, participants were recruited from the Drexel University Comprehensive NeuroAIDS Center (CNAC) cohort (supported by NIMH P30MH092177) over an 8-month period (November 2020–May 2021). Following a chart review, PWH on antiretroviral therapy, with a previous comprehensive neuropsychological assessment (CNPA), and previous CNAC research experience, were invited to participate. Study participants were recruited and screened by a telephone interview and provided verbal consent. Table 1 depicts the study’s exclusion criteria, which were selected to minimize confounding factors and isolate HAND-specific neuropsychological impairment (Tedaldi et al., 2015). Having a valid driver’s license was not used as an exclusion criterion, given the sociodemographic characteristics of the CNAC cohort (urban, low income, where suspended or expired driver’s licenses are more common, but driving is not a foreign activity). All study procedures were approved by the Drexel University Institutional Review Board and were completed on Drexel University School of Medicine premises (Philadelphia, Pennsylvania).

**TABLE 1:** Study exclusion criteria.

Exclusion criteria	Method to ascertain	Ascertained during	Rationale
Children under age 18 years	Medical chart review	Pre study visit	Drexel University’s HIV clinic typically does not see children
Diagnosis of neurocognitive impairment for reasons other than HIV (e.g., Alzheimer’s, Parkinson, etc.)	Medical chart review	Pre study visit	Minimize confounding factors and isolate HAND-specific neuropsychological impairment
Positive for illegal narcotics use at time of study visit (opioid or cocaine use)	Urine drug screen examination	Study visit	Minimize confounding factors and isolate HAND-specific neuropsychological impairment
Currently pregnant	Self-report	Study visit	Risk factor for simulator-based motion sickness
History of migraine headaches	Self-report	Study visit	Risk factor for simulator-based motion sickness
Not driving a motor vehicle within the last 12 months	Self-report	Study visit	Minimize confounding due to no driving experience or knowledge

### Study visit procedures

Clinical eligibility criteria were ascertained prior to the telephone interview *via* a medical chart review. The telephone interview was used to confirm eligibility criteria (except for the urine drug screen), obtain verbal consent, and schedule the study visit. During the study visit, written informed consent was obtained and participants were then asked to complete a urine drug screen examination. Participants that tested positive for cocaine or illegal opioid use were excluded from subsequent study visit procedures. Marijuana (medical or recreational) use was not an exclusion criterion as its use is very prevalent in the community. Additionally, marijuana use can yield a positive urine test for up to 30 days, whereas a positive result for other drugs of abuse typically indicates more recent use (e.g., in the past 3 days for cocaine). All other participants proceeded to complete a self-report survey (to collect driving history and other demographic information), a CNPA, and finally, the VDT.

### Administering the comprehensive neuropsychological assessment

The CNPA (lasting approximately 1.5–2 h) was administered by a trained psychometrist (blinded to all other study data collected) to confirm the presence or absence of HAND through an assessment of seven domains, using at least two measures per domain (Table 2) in accordance with Frascati criteria (Antinori et al., 2007). Tests were selected for their sensitivity to HAND (Carey et al., 2004a,b; Woods et al., 2004; Monteiro de Almeida et al., 2016).

**TABLE 2:** Component tests of the CNPA.

Domain assessed	Component tests
Processing speed	Wechsler Adult Intelligence Scale—4th Ed. (WAIS-IV) Coding Trail Making Test—Part A Stroop Word and Stroop Color
Attention and working memory	WAIS-IV Digit Span: Forward, Backward, and Sequencing
Motor function	Grooved Pegboard Test: Dominant and non-dominant
Executive function	Trail Making Test—Part B Stroop Color Word Modified Wisconsin Card Sorting Test (M-WCST) Errors
Language	Letter fluency (FAS) Animal fluency Boston naming test-30
Verbal memory	Hopkins Verbal Learning Test—Revised (HVLT-R): Immediate Recall, Delayed Recall, and Recognition
Visuospatial memory	Brief Visuospatial Memory Test—Revised (BVMT-R): Immediate Recall, Delayed Recall, and Recognition

### Administering the virtual driving test

The VDT (*Ready-Assess*™; ready-assess.com) is a well-established driving assessment platform used in both commercial and clinical settings (Walshe et al., 2020; Lee, 2021). In a self-directed workflow (containing both voiceover prompts and on-screen navigational instructions), study participants were exposed to ecologically valid crash scenarios (McDonald et al., 2014) contained in a typical driving route, lasting approximately 15 min. Upon completing a brief eye-tracking calibration step (Tobii 5^1^), study participants were then asked to independently complete the VDT by logging into the VDT workstation with their assigned research identification number. Designed to be installed in a limited-space environment (e.g., a small office desk), the VDT workstation included an internet-connected Windows PC, a Logitech G29 (steering wheel and pedals) and a pair of over-the-ear headphones. Upon logging into the VDT software installed on the workstation, each participant was exposed to three primary modules in a self-directed manner. In the first module, an animated orientation video was presented, lasting approximately 30 s. The purpose of this video was to provide the participant a basic introduction to the VDT. The second module incorporated an introductory drive. During this module, the participant was introduced to the different VDT control inputs (e.g., steering, brake, throttle, turn signal, transmission, etc.). Additionally, the participant was asked to complete a series of basic driving maneuvers (accelerating, braking, steering through a curve and executing a 90-degree turn) to acclimate themselves to the program. This module lasted approximately 3 min and allowed the participant to repeat the process if needed. The final module was a virtual driving test route which was scored for performance. The route contained variations of common crash scenarios as described in McDonald et al. (2014) and common driving tasks and roadway hazards (e.g., pedestrian crosswalks, school zones, ambulances, etc.), embedded in a typical driving route, lasting approximately 10 min. Each participant was exposed to the same VDT driving route (same tasks and exposures in the same order).

### Data preparation

#### Confirming the presence of HIV-associated neurocognitive disorders

Raw, unadjusted scores from the individual CNPA tests were converted to demographically adjusted (age, gender, education, and race) T-scores using published norms based on HIV-seronegative healthy populations (Strauss et al., 2006; Norman et al., 2011; Schretlen et al., 2018). T-scores for each test were then converted to deficit scores, and a deficit score for each domain (average of deficit scores for all tests in the domain) and a Global Deficit Score (GDS: average of domain deficit scores) were computed for each participant using established methods (Carey et al., 2004a; Blackstone, 2012). A GDS cutoff of ≥ 0.5 was used to indicate the presence of HAND (Carey et al., 2004a). In addition to HAND status, a cutoff of ≥ 1 was applied to domain deficit scores to indicate the presence of impairment in that domain. Standard three-level GDS-defined HAND categories (Antinori et al., 2007) were also computed, however, were not used in the primary analysis due to HIV-associated dementia being relatively rare in this cohort. The GDS approach to HAND classification was utilized because recent evidence demonstrates that GDS-based HAND diagnoses are more specific than Frascati criteria-based diagnoses involving the assessment of functional impairments of ADL (Heaton, 2021).

#### Virtual driving test data processing

Each VDT session generated a JSON file (referred to as a replay file) containing a 10 Hz multivariate, time series record. The replay file was used to compute 40 unique time series channels to measure driving performance across multiple driving elements (e.g., speed, lane deviation, following distance, elapsed time). Within the 40 time series channels, 8 eye-tracking channels were computed (using an automated, time-synchronized integration procedure developed by Diagnostic Driving, Inc.) to quantify eye-gaze movement during the VDT session. These channels were ultimately converted into unique variables (e.g., min, max, mean, median, standard deviation of each channel) and further organized into global (across the entire VDT route) and zone-specific variables (within 22 unique segments of the driving route). In total, 2,921 time-series-derived variables were computed for each study participant’s VDT session (previously described in Grethlein et al., 2020). In addition to the variables described above, 8 higher-order errors (e.g., counts of collisions, red light errors, stop sign errors, navigational errors, etc.) were automatically recorded in the replay file to compute a composite score of VDT performance (*VDT Error Score*) based on a linear combination of these higher-order error counts. This composite score of performance has been previously used to predict on-road examination results for licensure (Winston et al., 2019). In total, a completed VDT session yielded 2,601 explanatory variables, including *VDT Error Score* (after removing variables with zero variance). The relatively large number of variables with zero variance was expected due to the number of unique, evaluable zones along the VDT driving route (e.g., we would expect zero pedestrian collisions in a zone without pedestrians). Additionally, it is acknowledged that several multi-collinear relationships exist among these variables. No additional data processing was conducted (scaling of variables or removal of outlier cases) prior to analysis.

### Final analytical dataset and analytical methods

A final analytical dataset consisting of 2,601 explanatory variables and one binary outcome variable for HAND status was generated, yielding a matrix of size *n*x2602, where *n* would be the final sample size. In addition, seven binary variables were generated to indicate impairment within each of the seven CNPA domains.

#### Summary of supervised variable selection methods used

Given the small sample size and high dimensionality of the final dataset, systematic reduction methods were required to identify which VDT variables, if any, were associated with HAND. Our strategy was to utilize a methodology that would allow for maximum interpretability of results (as the goal of the VDT is designed to provide practical guidance on safe driving), thereby ruling out common reduction techniques such as principal component analysis that transform input variables into a potentially less intuitive space by aggregation of variables.

**FIGURE 1:**
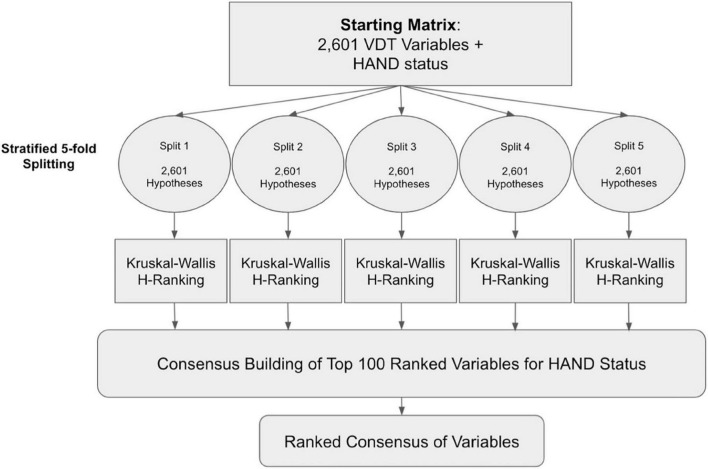
Analytical workflow for dimensionality reduction via a two-stage variable selection.

In order to overcome the multiple comparisons problem for this small dataset, our analysis leverages re-samplings of the dataset similar to stratified fivefold cross-validation: splitting the labeled samples into 5 disjoint folds, ideally preserving the ratio of samples with and without HAND in each split. Splitting the dataset combined with non-parametric Kruskal-Wallis (KW) tests, was used to provide five opportunities to independently test each of the 2,601 individual hypotheses and then rank each variable during a two-stage variable selection process (using the KW *H*-value, see Figure 1). Specifically, we generated five different training sets (but we did not make use of the five test sets) to alleviate concerns around identifying false-positive variables (a limitation of multiple hypothesis testing).

With these five training sets, we obtained five independently generated lists of selected VDT performance variables; cross-examining which variables were found to be consistently associated with HAND in all five splits to build a consensus. A random seed set to 0 was used for the stratified splitting procedure (for reproducibility). Additionally, the stability and robustness of variable selection results were evaluated using “top k-Lists” normalized Kendall rank correlation (*p* = 0.5 “neutral approach”; Fagin et al., 2003) and Jaccard similarity (Saeys et al., 2008), to demonstrate that variables were not selected due to random chance or overfitting to a sub-sample of participants produced from splitting. All analyses were conducted using Python (version 3.8.9)^2^ and R (version 4.0.2).^3^

#### Variable selection and forming a final consensus

Using the training data (a unique subset of fourfold of VDT data) in each split, we computed the univariate KW *H*-value for each of the 2,601 VDT variables relative to HAND status. The VDT variables in each split were then ranked in descending order of univariate KW *H*-value. In all experiments conducted, the “top ranked variable” would have a rank of 1 (inferring the VDT variable most associated with HAND status), and the lowest ranked VDT variable would have a rank of 2601. Ranking ties were handled by deferring to the VDT variable that appeared first in the original dataset (from the start of the drive). In each split we extracted the “top k variables” ranked list of selected VDT variables.

By experimental design, the “top k variables” selected in one split were not dependent on the “top k variables” selected from any other split, though the training data from any two splits shared roughly 50% of the same VDTs (twofold of overlap, similar to fivefold cross-validation). A second-stage filtering criterion was imposed so that only VDT variables that were consistently an element of the “top k variables” ranked lists in all 5 splits would be included in the consensus, i.e., the consensus was formally defined as the set intersection of the 5 “top k variables” ranked lists. For each VDT variable in the consensus, we computed the median KW *H*-value across the 5 splits, estimating how associated an individual VDT variable was in at least three of the five splits. These median KW *H*-values were used to rank the consensus in descending order. There was no guarantee that such an intersection would be non-empty, as the likelihood of any single VDT variable being consistently ranked in the “top k variables” in all 5 splits drops as k decreases.

To choose k prior to producing the final rankings, we tested integer values of k ∈ [1, 1,000] as thresholds for forming consensus using the KW *H*-value as the ranking score. By monitoring the mean KW *H*-Value of the “top k variables” selected in each split, we evaluated the relative trade-off between selecting a smaller number of supposedly informative VDT variables and balancing the diminishing returns of including a greater number of less informative variables into the selected group. We used the “kneed” python package (Satopa et al., 2011) to find the “elbow point” value k for each split to estimate the exact value of k where the trade-off stops working in our favor. In most cases, the “elbow point” for the value of k was between 150 and 200 (results omitted for brevity). Using the “elbow point” results to guide our choice of k, we chose to conservatively build our consensus using the *k* = 100 top ranked VDT variables from all five splits for the HAND outcome variable. This was done to cast a wide enough net to allow for some VDT variables to make it into the consensus, while using intermediary ranking lists of constant length that are not so large as to allow uninformative VDT variables into the consensus.

#### Final variable categorizations

We utilized the authorship team’s subject matter expertise to review the final ranked-consensus list of VDT variables and assign each variable to a measured driving domain(s). Furthermore, and as part of our secondary aim, each variable in the final subset was evaluated to measure its relative association with impairment in each of the seven cognitive domains (independently assessed during the CNPA; Table 2) using a Spearman correlation matrix with a Bonferroni correction.

## Results

### Final analytical sample and characteristics

Seventy-nine (79) participants scheduled a study visit after providing verbal consent. Among these, 9 did not show up for their study visit and 1 participant was further excluded from subsequent procedures after testing positive during their urine drug screen examination. In addition, 1 participant declined to participate in subsequent study procedures after their negative urine drug screen (did not complete CNPA or VDT), 1 participant had an invalid CNPA, 1 participant could not complete the VDT due to feeling dizzy, and 4 participants could not complete the VDT due to network or equipment issues. A final study sample of sixty-two (62) participants met all study eligibility criteria and completed all study visit procedures. Among the final 62 participants, 37% (*n* = 23) tested positive for allowable drug use (prescribed pain management, psychiatric medication, or marijuana—common in this population). Of note, compared to participants who tested negative for allowable drug use, participants who tested positive, did not perform differently in the VDT (as measured by *VDT Error Score*; *W* = 404; *p* = 0.52) and did not have a different prevalence of HAND (χ^2^ = 7.3*10^–5^; *p* = 0.99). The demographic and clinical characteristics of the final study sample are detailed in Table 3 and generally matched the overall characteristics of the entire CNAC cohort.

**TABLE 3:** Demographic and clinical characteristics of the final study sample.

Characteristic	Result (*N* = 62)
Age (years)	
Mean (SD)	54.4 (9.3)
Gender	
Male Female	69.4% 30.6%
Race	
Black/African American White	93.5% 6.5%
Driver’s license status	
Current and valid Suspended or revoked Permit only No current license	71.0% 13.0% 4.8% 11.2%
*Driving history*	
Drove in the past 12 months Did not drive in the past 12 months	100.0% 0.0%
Latest viral load (copies/mL)	
<200 ≥200	87.1% 12.9%
Nadir CD4 count (cells/μL)	
<200 ≥200 Mean (SD)	38.7% 61.3% 289.0 (149.9)

More than half of the final study sample (*N* = 35; 57%) had a confirmed presence of HAND, as determined by the CNPA. Among those with HAND, 83% (*n* = 29) had either asymptomatic or mild impairment, and 17% (*n* = 6) had severe impairment (HIV-associated dementia). The prevalence of impairment within the seven cognitive domains assessed during the CNPA is detailed in Table 4. There were no statistically significant differences between having a HAND diagnosis and the demographic and clinical characteristics of the study participants. There were statistically significant differences between *VDT Error Score* and these characteristics. However, not having a current and valid driver’s license was significantly associated with having HAND [Note: Pennsylvania is a mandatory medical reporting state for driving privileges (Pennsylvania Department of Transportation, 2022)].

**TABLE 4:** Prevalence of within-domain impairment among the study participants.

	*% Domain deficit score* > *0*			
Domain	All participants (*N* = 62)	Presence of HAND (*N* = 35)	Absence of HAND (*N* = 27)	*P*
Processing speed	14.5%	20.0%	7.4%	0.28
Attention and working memory	24.2%	40.0%	3.7%	<0.01
Motor function	38.7%	58.8%	14.8%	<0.01
Executive function	14.5%	25.7%	0%	<0.01
Language	11.3%	20.0%	0%	0.02
Verbal memory	54.8%	80.0%	22.2%	<0.01
Visuospatial memory	38.7%	54.3%	18.5%	0.01

Exact tests were used to examine differences among those with and without HAND.

### Variable selection results

Using the fivefold consensus approach, we were able to identify a final subset of 20 VDT variables that were associated with HAND status (Table 5). The consensus identified a mixture of six global (across the entire VDT) variables and fourteen zonal (within specific segments of the VDT route) variables. Of particular interest, the composite score of cumulative serious errors, *VDT Error Score*, was identified in the final subset (median *H*-value of 7.33, fivefold median *p* = 0.007) and was statistically associated with impairment of motor function, executive function, attention and working memory, and verbal memory. VDT performance variables that were statistically associated with a cognitive domain before or after the Bonferonni correction are reported in Table 5.

**TABLE 5:** The final 20 ranked consensus variables associated with HAND status.

Rank	VDT variable description	Type	Driving domain(s) assessed	Cognitive domains that were statistically associated with the VDT variable in this analysis (Note: Bolded domains indicate statistical significance maintained after a Bonferroni correction for multiple comparisons)
1	Mean forward jerk while traversing an inactive railroad crossing	Zonal	*Primary*: Vehicle control *Secondary*: Hazard anticipation and response	Language
2	Number of collisions with vehicles from sides or behind	Global	*Primary*: Vehicle control	Attention and working memory Motor function **Executive function** Language Visuospatial memory
3	Maximum lane deviation of vehicle driving along a curve with an active school playground on the right side	Zonal	*Primary*: Road positioning *Secondary*: Hazard anticipation and response	**Attention and working memory** Executive function
4	Median lane deviation of driver vehicle traversing a 4-way traffic-light-controlled intersection instructed to make a left turn	Zonal	*Primary*: Road positioning *Secondary*: Gap selection Communication and right of way	Attention and working memory
5	Mean forward acceleration while traversing an inactive railroad crossing	Zonal	*Primary*: Vehicle control *Secondary*: Hazard anticipation and response	Language
6	Number of collisions with other vehicles (all orientations of vehicle-to-vehicle collisions).	Global	*Primary*: Vehicle control	Attention and working memory **Motor function** Executive function Verbal memory
7	Number of collisions with static objects	Global	*Primary*: Vehicle control	Motor function Executive function
8	*VDT Error Score* (a linear combination of serious error counts along the entire VDT route)	Global	*Primary*: Vehicle control Hazard anticipation and response Gap selection Speed management Communication and right of way Attention maintenance	Attention and working memory **Motor function** Executive function Verbal memory
9	Minimum difference of vehicle speed relative to posted speed limit while traversing a speed-reduction construction zone	Zonal	*Primary*: Speed management *Secondary*: Hazard anticipation and response	Motor function Executive function Verbal memory Visuospatial memory
10	Minimum forward acceleration while traversing an inactive railroad crossing	Zonal	*Primary*: Vehicle control *Secondary*: Hazard anticipation and response	Attention and working memory **Verbal memory**
11	Maximum forward jerk of vehicle while traversing an inactive railroad crossing	Zonal	*Primary*: Vehicle control *Secondary*: Hazard anticipation and response	Attention and working memory Verbal memory
12	Standard deviation of heading misalignment with road following direction while traversing a 4-way traffic-light-controlled intersection instructed to make a right turn	Zonal	*Primary*: Road positioning *Secondary*: Gap selection Communication and right of way	Attention and working memory Motor function Executive function
13	Minimum signed distance from road center (negative values indicate driving on the left side of the road, potentially into oncoming traffic, over the median)	Global	*Primary*: Road positioning	Attention and working memory **Motor function** Executive function
14	Standard deviation of steering wheel while an active-moving ambulance approaching from behind	Zonal	*Primary*: Road positioning *Secondary*: Vehicle control Hazard anticipation and response Communication and right of way	Attention and working memory Motor function Executive function
15	% Time driven ≤ 15 mph below posted speed limit while traversing a traffic-light-controlled 4-way intersection instructed to make a left turn onto main arterial	Zonal	*Primary*: Speed management *Secondary*: Gap selection Communication and right of way	Attention and working memory Motor function Executive function Verbal memory
16	Number of vehicle teleports (vehicle returned back to a starting position after a significant deviation from the planned route)	Global	*Primary*: Attention maintenance	Attention and working memory Motor function Executive function
17	Minimum steering wheel rotation while an active-moving ambulance approaching from behind	Zonal	*Primary*: Road positioning *Secondary*: Vehicle control Hazard anticipation and response Communication and right of way	Attention and working memory Visuospatial memory
18	% Time driven ≤ 10 mph below posted speed limit while traversing an 4-way stop sign-controlled intersection, instructed to make a right turn	Zonal	*Primary*: Speed management *Secondary*: Gap selection Communication and right of way	**Attention and working memory** **Executive function** Verbal memory
19	Maximum heading misalignment with road following direction while an active-moving ambulance approaching from behind	Zonal	*Primary*: Road positioning *Secondary*: Hazard anticipation and response Communication and right of way	Processing speed Attention and working memory Motor function Executive function Language
20	Mean heading misalignment with road following direction while traversing a speed-reduction school zone	Zonal	*Primary*: Road positioning *Secondary*: Hazard anticipation and response	Attention and working memory Executive function

The distributions of each of the 20 identified VDT variables was examined and described in Table 6 to identify practical differences between participants with and without HAND.

**TABLE 6:** Rank-ordered distributions of the 20 identified VDT variables.

Rank	VDT variable units	All cases (*N* = 62)		HAND: Present (*n* = 35)		HAND: Absent (*n* = 27)		Abs% median difference	Fivefold median KW *H*-value	Fivefold median KW *p*-value
		*Median*	*IQR*	*Median*	*IQR*	*Median*	*IQR*			
1	Mph/sec^2^	−1.14	13.60	−4.52	8.40	3.81	13.56	184%	10.06	0.002
2	Count	0.00	1.00	0.00	1.00	0.00	0.50	UNDEF	9.52	0.002
3	Meters	1.73	0.50	1.74	0.04	1.36	0.69	22%	9.42	0.002
4	Meters	0.58	0.51	0.75	0.40	0.38	0.33	49%	9.06	0.003
5	Mph/sec	0.19	1.72	0.59	1.07	−0.45	1.77	176%	8.94	0.003
6	Count	0.50	2.00	1.00	2.00	0.00	1.00	100%	8.43	0.004
7	Count	1.00	1.75	1.00	3.00	0.00	1.00	100%	7.55	0.006
8	Error Point	32.50	36.50	46.00	66.50	27.00	26.00	41%	7.33	0.007
9	Mph	−35.05	9.42	−28.49	14.97	−35.10	0.25	23%	7.33	0.007
10	Mph/sec	−2.65	6.41	−2.13	2.64	−4.78	6.98	124%	7.22	0.007
11	Mph/sec^2^	22.24	51.54	18.22	20.06	38.92	64.42	114%	7.17	0.007
12	Degrees	0.99	1.34	1.30	1.64	0.58	0.54	55%	7.17	0.007
13	Meters	−4.17	4.75	−6.47	8.08	−3.48	2.58	46%	7.11	0.008
14	% Rotation	0.01	0.01	0.02	0.02	0.01	0.01	50%	6.90	0.009
15	% Time	0.87	0.31	0.77	0.41	0.98	0.16	27%	6.79	0.009
16	Count	0.00	0.75	0.00	1.00	0.00	0.00	UNDEF	6.44	0.011
17	% Rotation	−0.02	0.02	−0.03	0.02	−0.02	0.01	33%	6.33	0.012
18	% Time	1.00	0.00	1.00	0.14	1.00	0.00	0%	6.17	0.013
19	Degrees	3.46	2.92	4.21	3.40	2.83	1.90	33%	6.08	0.014
20	Degrees	1.78	1.90	1.99	2.22	1.53	1.12	23%	5.14	0.023

### Stability of HIV-associated neurocognitive disorders rankings

**FIGURE 2:**
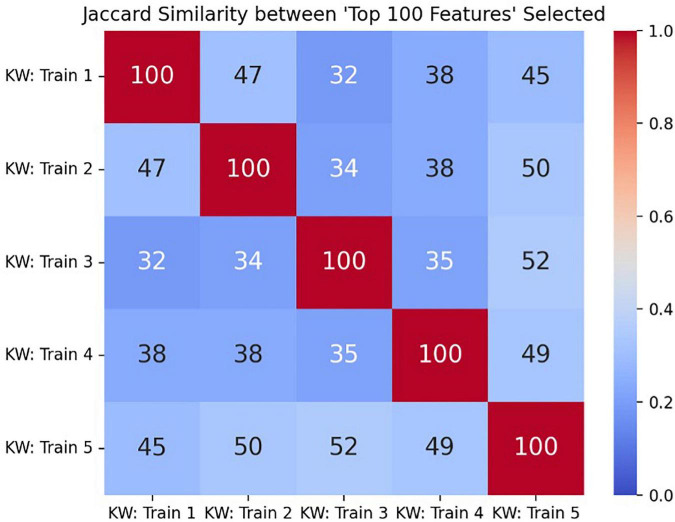
The Jaccard similarity score comparing all pairs of “top 100 variables” rankings lists, with the true size of the intersection (number of shared VDT performance variables selected in both intermediate rankings lists) annotated in each cell.

On average, any generated pair of “top 100 variables” rankings lists shared 42 VDT variables in common (average Jaccard similarity of 0.27) and had a normalized Kendall correlation value of less than 0.15. Both quantities indicated that similarly ranked subsets of VDT variables were consistently selected as part of the “top 100 variables” intermediate rankings. If the probability for any single VDT variable being selected in the “top 100 variables” rankings list was uniformly distributed across all 2,601 variables, then any given variable had a probability of 8.40*10^–8^ of being selected independently in five such ranked lists. Figure 2 depicts comparisons of the “top 100 variables” rankings from all five splits utilized.

## Discussion

This study presents an initial examination of VDT performance among PWH and its relationship with HAND and impairment within seven domains assessed during the CNPA. Applying a non-traditional and systematic data reduction method to a novel application, we were able to identify a smaller and stable subset of VDT performance variables, derived from a consensus generation process. Reducing the dimensionality of the dataset (*via* variable selection), allowed us to parsimoniously measure VDT performance and its relationship with HAND. Additionally, stability results provide further support that the variables were not selected due to random chance or overfitting to a sub-sample of participants. Rather, the stability results indicate that the consensus represents a reliable subset of VDT variables that could aid in the detection of individuals with HAND. Using this approach, 20 VDT variables were identified in the subset, representing a range of variables across the entire VDT route (global) and within specific regions and tasks along the route (zonal). The identification of several variables measuring vehicle control, road positioning, attention maintenance, and hazard anticipation and response corroborate previous findings examining deteriorated driving performance among individuals with HAND (Marcotte et al., 1999, 2004, 2006; Foley et al., 2013; Vance et al., 2014; Gouse et al., 2021). Furthermore, the inclusion of the composite *VDT Error Score* in the final consensus provides initial evidence that the VDT can assess complex, cognitive functioning related to HAND. Participants with HAND had nearly twice the *VDT Error Score* than those without HAND, suggesting that the level of complex cognitive and sensorimotor control required to drive safely and avoid collisions was manifested in VDT performance. Individual zones such as the “playground curve” (left-leaning curved roadway with an active school playground on right side) and “railroad zone” (congested roadway with an inactive railroad crossing) were designed to simultaneously test a variety of safe driving domains (e.g., vehicle control, road positioning, hazard anticipation, and response). Results in Table 5 demonstrate that these complicated “zonal” scenarios within the VDT can also tax the complex cognitive control implicated in HAND, as the level of cognitive and sensorimotor control required to safely traverse these zones is diminished in participants with HAND (greater lane deviation, heading misalignment and distance from roadway center events). In this study, as an initial step toward developing a valid screen for HAND, we identified a group of VDT variables (and their underlying tasks) within the domains of motor function, executive function, and attention and working memory that support the use of the VDT platform for identifying clinically significant functional impairment associated with HAND. Our findings provide preliminary evidence that the VDT can probe complex cognitive and sensorimotor control involved with HAND and potentially serve as a low-cost screening tool to detect HAND earlier in the disease course. However, additional research is required to develop and validate a predictive HAND screening model incorporating the subset of VDT variables identified from this analysis.

### Limitations

Our results are not without limitations. We did not include a healthy control group in the analysis as a mechanism to compare VDT performance among PWH who do not have HAND with a healthy population (HIV-negative and no neurocognitive impairment). Second, the ratio of sample size to evaluable variables was low. Our evaluation of “top 100 variables” ranking stability indicated that even within a small number of participants, there was significant variance in VDT performance in regards to HAND status. Third, we did not utilize “traditional” data reduction methods; rather, we designed our analytical methods to produce interpretable results (rankings of the VDT variables relative to HAND status). Fourth, our variable selection method did not identify any visual scanning variables (important in the evaluation of safe driving performance). This could be due to our limited sample size and our strict criteria for variable selection. Visual scanning metrics did appear in a final consensus of variables when restrictions on variable selection were eased. Fifth, the order of administering the CNPA and the VDT during the study visit was not randomized. All participants completed the CNPA before the VDT. This was due to clinical workflow constraints and the availability of the psychometrist. Sixth, 22% (*n* = 17) of the 79 participants who scheduled a study visit, did not complete all study procedures. Of these, the majority (*n* = 10; 59%) did not show for their study visit or declined study visit procedures. This is a common logistical limitation of conducting clinical research in this population. Finally, this study excluded individuals who tested positive for illegal substance abuse in order to better isolate HAND-specific impairment and test our primary hypotheses. Illegal substance abuse may be common among PWH and impact the broader applicability of using the VDT as a screen for HAND in this population. Future studies will incorporate participants who use illegal substances in order to better understand how it impacts VDT performance and its relationship with HAND.

## Conclusion

This study identified a subset of VDT performance variables that are associated with HAND. The current findings have important implications for developing a new screening paradigm for HAND: using the VDT platform as a clinically feasible and ecologically valid assessment of function. However, additional research is needed to develop and validate a HAND screening model incorporating the final subset of VDT variables identified.

## Data availability statement

The dataset presented in this article is not readily available because this research was funded by a Small Business Innovation Research grant (SBIR) from the National Institutes of Health (NIH) and awarded to Diagnostic Driving, Inc., with the specific purpose of commercializing intellectual property generated from this research program. Requests to access the dataset should be directed in writing to Diagnostic Driving, Inc. by DG, david@diagnosticdriving.com.

## Ethics statement

The studies involving human participants were reviewed and approved by the Drexel University Institutional Review Board. The patients/participants provided their written informed consent to participate in this study.

## Author contributions

DG: responsible for VDT performance variables ranking analysis and overall manuscript preparation. VP: critical review and data collection methods. KD: CNPA methods and critical review. WD: CNPA methods and critical review of analytical methods. ZS, EW, DK, BA, KM, ST, BW, and FW: critical review. SO: critical review of analytical methods. VK: critical review and overall manuscript preparation. All authors contributed to the article and approved the submitted version.

## Footnotes

1
gaming.tobii.com

2
python.org

3
r-project.org
